# Small Extracellular Vesicles Derived from Adipocytes Attenuate Intervertebral Disc Degeneration in Rats by Rejuvenating Senescent Nucleus Pulposus Cells and Endplate Cells by Delivering Exogenous NAMPT

**DOI:** 10.1155/2021/9955448

**Published:** 2021-08-14

**Authors:** Yongjin Sun, Xu Li, Xiaoxu Yang, Bi Chen, Wenzhi Zhang

**Affiliations:** ^1^Spine Center, Department of Orthopaedics, The First Affiliated Hospital of USTC, Division of Life Sciences and Medicine, University of Science and Technology of China, Hefei, Anhui 230001, China; ^2^Department of Orthopedics (Spine Surgery), First Affiliated Hospital of Wenzhou Medical University, Wenzhou 325000, China

## Abstract

Cellular senescence is a key factor in the development of intervertebral disc degeneration (IVDD). Age-associated decreases in NAD^+^ levels play a critical role in regulating cellular senescence. Previous studies have found that small extracellular vesicles (sEVs) secreted by adipocytes (Adipo-sEVs) or adipose tissue are abundant in nicotinamide phosphoribosyltransferase (NAMPT), which is the key NAD^+^ biosynthetic enzyme in mammals. Systemic injection of these sEVs significantly improves physical activity and extends the lifespan of aged mice by increasing NAD^+^ levels. However, to date, the therapeutic potential of Adipo-sEVs in other age-associated disease models, such as IVDD, has not been explored. In this study, we investigated the therapeutic effects of Adipo-sEVs on senescence of nucleus pulposus cells (NPCs) and cartilaginous endplate cells (EPCs). In vitro, Adipo-sEVs could rejuvenate the senescence of NPCs and EPCs. Age-related dysfunctions were also ameliorated by Adipo-sEVs by delivering NAMPT and activating NAD^+^ biosynthesis and the Sirt1 pathway. Further in vivo experiments revealed that Adipo-sEV-mediated delivery of NAMPT attenuated IVDD in rats by rejuvenating senescent NPCs and EPCs. Collectively, the results indicate a new cell-free tool and provide a promising sEV-mediated delivery method of NAMPT as a therapeutic approach for IVDD clinically.

## 1. Introduction

Intervertebral disc degeneration (IVDD) is widely thought to contribute to a series of painful spinal disorders and poses a major global health threat [1]. The intervertebral disc (IVD) comprises the outer annulus fibrosus (AF), inner nucleus pulposus (NP), and up-down cartilaginous endplate (EP). AF and NP allow the disc to manage diverse external mechanical stresses, while EP is crucial for the inner NP nutrient supply. Most nutrients for NP diffuse through the EPs because IVDs have limited blood vessels [2]. Accumulating evidence indicates that the senescence of NP cells (NPCs) and EP cells (EPCs) plays a pivotal role in the development of IVDD [3, 4]. Oxidative stress in IVD may be caused by external overload drive cells to exhibit aging-associated phenotypes [5, 6]. Senescent NPCs show distinct catabolic features characterized by the reduced synthesis of constituents of the extracellular matrix (ECM), such as proteoglycan and collagen II, and increased secretion of the senescence-associated secretory phenotype (SASP), which play significant roles in IVDD [7]. However, senescent EPCs exhibit excessive calcification. This leads to blockage of the nutrition channel in EP and disrupts the balance of nutrients and metabolites entering and exiting the disc, which further accelerates the degeneration of NPCs [8, 9]. Therefore, therapeutic strategies aimed at rejuvenating the senescence of NPCs and EPCs should effectively prevent or reverse the progression of IVDD.

For the past few years, increasing studies have indicated that nicotinamide adenine dinucleotide (NAD^+^) may play a critical role in regulating aging [10]. Increased NAD^+^ consumption and decreased NAD^+^ biosynthesis would result in various age-associated pathological progress in multiple tissues. NAD^+^ levels in many tissues, such as those of the neurosensory retina, brain, skin, skeletal muscle, and liver, decline with age, and restoring NAD^+^ levels could dramatically counteract aging [11]. The reduction of NAD^+^ may be the crucial event in the progression of age-associated pathology. Therefore, developing an effective strategy to increase NAD^+^ biosynthesis is a potential approach for antiaging intervention. Recent research has found that small extracellular vesicles (sEVs) secreted by adipose tissue or adipocytes are abundant in nicotinamide phosphoribosyltransferase (NAMPT), which is the key NAD^+^ biosynthetic enzyme in mammals [12]. sEVs are a class of natural nanoparticles enclosed by a lipid bilayer with diameter of 30-150 nm. sEVs can transport their cargos of parental cells, including nucleic acids and proteins, into recipient cell. Systemic injection of NAMPT-containing sEVs significantly delays aging and extends the lifespan of aged mice. NAMPT-containing sEVs derived from adipocytes promote systemic NAD^+^ biosynthesis [12, 13]. Their results also suggested that extracellular NAMPT is mainly encapsulated by sEVs for delivering. Moreover, the application of sEVs avoids several shortcomings of adipocyte transplantation, such as an undesirable immune response and inconvenient storage [14]. As a consequence, sEVs derived from adipocytes (Adipo-sEVs) are a promising strategy for treating age-associated diseases. However, to date, the therapeutic potential of Adipo-sEVs for IVDD, especially for senescence of NPCs and EPCs, has not been evaluated.

In this study, we harvested adipocytes from the HS-5 cell line, a human bone marrow stromal cell line, and purified sEVs from the culture media of HS-5 adipocytes. We then tested the therapeutic effects of Adipo-sEVs on senescent NPCs and EPCs and explored the underlying mechanisms. Our results showed that Adipo-sEVs could deliver NAMPT to senescent NPCs and EPCs and enhance NAD^+^ biosynthesis in cells. Consequently, the senescent NPCs and EPCs were rejuvenated, and age-related dysfunction was ameliorated in vitro. Further in vivo experiments showed that Adipo-sEVs significantly delayed the development of IVDD in rats and alleviated cellular senescence in NP and EP. These results indicate a promising therapeutic strategy for IVDD.

## 2. Materials and Methods

### 2.1. Cell Isolation and Culture

The HS-5 cell line was incubated in high-glucose DMEM (Gibco) supplemented 10% foetal bovine serum (FBS; Gibco) and penicillin/streptomycin (1%) under 37°C and 5% CO_2_. HS-5 cells were fully differentiated into adipocytes using lipogenetic differentiation medium (Cyagen Biosciences) according to the manufacturer's protocol. Human NPCs and EPCs were extracted from nucleus pulposus and cartilaginous endplate, respectively, using the methods described as previous studies [15, 16]. NPCs and EPCs were incubated in DMEM/F-12 medium (Gibco) supplemented 10% FBS and penicillin/streptomycin (1%).

### 2.2. Isolation and Identification of Adipo-sEVs

Adipo-sEVs were isolated by ultracentrifugation. Briefly, after HS-5 was fully differentiated into adipocytes, they were rinsed with PBS then cultured in serum-free lipogenetic differentiation medium for 48 h under 37°C. The conditioned medium was collected, and the sEVs were isolated using ultracentrifugation as previously described [12]. Transmission electron microscopy (TEM) was utilized to document the morphology of the sEVs. Nanoparticle analysis (NTA, ZetaView PMX 110) was used to detect the size distribution of the sEVs. The quality of isolated sEVs was analyzed by detecting the expressions of sEVs characteristic markers TSG-101 (Abcam), CD63 (Abcam), and CD9 (Abcam) and non-sEV marker GM130 (Abcam).

### 2.3. Lipogenetic Differentiation Assay

Adipocytes differentiated from HS-5 were identified using the Oil Red O staining kit (Beyotime) according to the protocol. Red fat droplets in adipocytes were observed by phase contrast microscopy.

### 2.4. Adipo-sEV Uptake Assay

To observe the uptake of sEVs by NPCs and EPCs, DiO fluorescent dye (Beyotime) was first used to label adipocytes. Then, the sEVs secreted by the DiO-labelled adipocytes were labelled with DiO. Next, DiO-labelled sEVs were added into conditioned medium and incubated with NPCs or EPCs for 12 h.

### 2.5. Western Blotting Analysis

We followed the methods of Sun et al. Briefly, total protein of cells was harvested using RIPA lysis buffer (Beyotime). Protein extracts were separated by using SDS-PAGE gels, followed by the transfer to a PVDF membrane. After blocking with 5% nonfat milk, the bands were incubated with indicated primary antibodies (Tsg101, Cell Signaling Technology; CD63, Abcam; CD9, Abcam; GM130, Abcam; NAMPT, Adipogen; P16, Abcam; MMP-3, Abcam; ADAMTS-4, Abcam; Aggrecan, Abcam; Collagen II Abcam; Sirt1, Abcam; Sirt3, Abcam; Sirt5, Abcam; *β*-actin, Abcam) overnight under 4°C. Then, the bands were incubated with horseradish peroxidase-linked anti-mouse IgG (Cell Signaling Technology) or anti-rabbit IgG (Abcam) secondary antibody for 1 h. The bands were exposed with enhanced chemiluminescence (ECL, Thermo Fisher Scientific).

### 2.6. SA-*β*-Galactosidase Staining

We followed the methods of Sun et al. Briefly, SA-*β*-Gal staining was done by using SA-*β*-Gal staining kit (Beyotime). The proportion of senescent cells, which were observed as blue-stained cells, was calculated under a phase contrast microscope.

### 2.7. Alizarin Red Staining

Calcification levels in EPCs were evaluated using an Alizarin Red staining kit (Beyotime). Briefly, EPCs from different treatment groups were fixed for 15 min, followed by wash twice with PBS, then the addition of Alizarin Red solution for 30 min, and then rinsing three times with PBS.

### 2.8. Immunofluorescence (IF) Analysis

IF staining was used to analyze Ki-67 (Affinity), Runx2 (Abcam), and Sirt1 (Abcam). Cells were fixed with 4% paraformaldehyde for 20 min under room temperature, followed by 0.25% Triton-X100 for 20 min, followed by blocking with 5% bovine serum albumin (Sigma) for 35 min, and finally incubation with indicated primary antibodies overnight under 4°C. Biotinylated secondary antibodies were incubated for 1 h under room temperature. The nuclei was counterstained with DAPI for 10 min.

### 2.9. Real-Time RT-PCR Analysis

We followed the methods of Sun et al. Briefly, total RNA was extracted from cells by using TRIzol Reagent (Invitrogen). Reverse transcription was done by using SuperScript II reverse transcriptase (Invitrogen). The real-time PCR analyze was done with the FastStart Universal SYBR Green Master.

### 2.10. NAD^+^ Level Assay

NAD^+^ levels of NPCs or EPCs from different treatment groups were detected using an NAD^+^ assay kit (Beyotime) according to the manufacturer's instructions.

### 2.11. Establishment and Treatment of the IVDD Model in Rats

Male S-D rats aged eight weeks were used for the in vivo experiments. All experimental procedures were permitted by the Animal Research Committee of the First Affiliated Hospital of USTC. A rat IVDD model was established as described previously [17]. One week after the initial surgery, 2 *μ*L of sterile saline (NS) with 1 × 10^10^ control Adipo-sEVs (CTRL-sEVs) or NAMPT-knockdown Adipo-sEVs (KD-sEVs)/mL was injected into the punctured level. The negative group received the same volume of 0.9% normal saline. The injection procedure was repeated every two weeks. MRI analysis was administrated on all rats eight weeks after initial surgery.

### 2.12. MRI

We followed the methods of Sun et al. Briefly, the degenerative changes of the punctured tail disc were evaluated in T2-weighted images by a 3.0 T clinical magnet (GE) after eight weeks of the puncture procedure. The Pfirrmann grading system was used to detect the classification of IVDD on T2-weighted images.

### 2.13. Histological Evaluation

All rats were euthanised, and the target tail samples were harvested and fixed, decalcified in 10% EDTA, embedded in paraffin, and cut into 5 *μ*m thick sections. Hematoxylin and eosin (H&E) staining was done for histological observation. The expression of P16 protein was evaluated by IF staining. The specimens were incubated overnight with indicated primary antibody (P16, Invitrogen), and incubated with secondary antibody conjugated to Alexa Fluor 488 (Abcam). The nuclei was counterstained with DAPI for 10 min.

### 2.14. Statistical Analysis

All data are displayed as the mean ± standard deviation (SD). Statistical analysis was conducted by utilizing one-way analysis of variance (ANOVA) or Student's *t*-test. A value of *P* < 0.05 was indicated statistically significance.

## 3. Results

### 3.1. Characterization of Adipocytes and Adipo-sEVs

**Figure 1:**
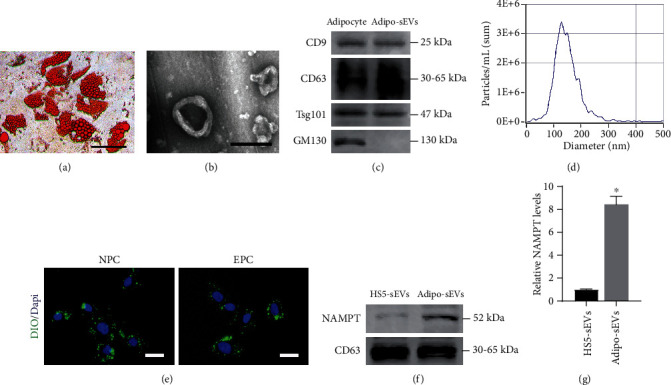
Characterization of adipocytes and Adipo-sEVs. (a) Oil red O staining of adipocytes differentiated from HS-5. (b) Morphology of Adipo-sEVs under TEM. Scale bar, 200 nm. (c) Western blotting analysis indicated that Adipo-sEVs expressed sEV markers, such as CD9, CD63, and TSG101, and were negative for GM130. (d) Particle size distribution of sEVs measured by NTA. (e) IF analysis of DiO-labelled Adipo-sEV internalization by NPCs and EPCs. Scale bar, 30 *μ*m. (f) Levels of NAMPT and CD63 in HS5-sEVs and Adipo-sEVs. (g) Densitometric quantification of the relative band intensity in (f). *n* = 3 per group. (^∗^*P* < 0.05 compared with HS5-sEVs).

Oil red O staining showed that the adipocytes were fully differentiated from HS-5 cells (Figure 1(a)), and we then characterized Adipo-sEVs. As shown in Figure 1(b), Adipo-sEVs exhibited a typical cup-shaped morphology. Western blotting analysis demonstrated that Adipo-sEVs expressed sEV markers, such as CD9, CD63, and TSG101, but were negative for GM130 (Figure 1(c)). NTA revealed that the particle size of the majority of Adipo-sEVs was approximately 80-200 nm (Figure 1(d)). Next, we determined whether Adipo-sEVs could be endocytosed into NPCs and EPCs. The results showed that DiO-labelled Adipo-sEVs were present in the perinuclear region after incubation for 12 h (Figure 1(e)). A previous study indicated that Adipo-sEVs encapsulate abundant NAMPT, which can be carried into cells and enhance NAD^+^ biosynthesis. Therefore, we also confirmed that fully differentiated adipocyte-derived sEVs contain the NAMPT protein (Figures 1(f) and 1(g)).

### 3.2. Adipo-sEVs Can Ameliorate the Senescent Phenotypes of NPCs and Age-Related Dysfunction In Vitro

**Figure 2:**
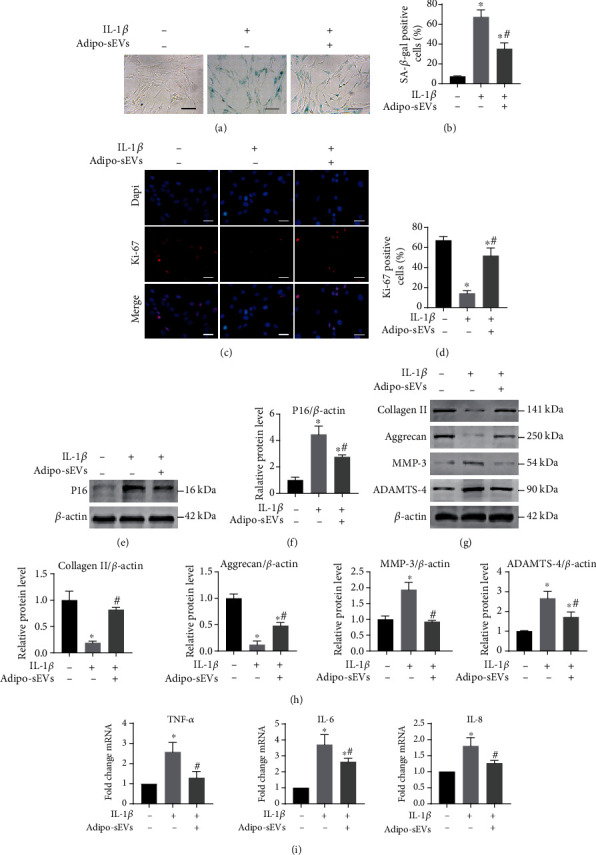
Adipo-sEVs can ameliorate the senescent phenotypes of NPCs and age-related dysfunction in vitro. (a) Representative micrographs of NPCs stained with SA-*β*-gal in different treatment groups. SA-*β*-gal-positive cells are shown in blue. Scale bar, 100 *μ*m. (b) Percentage of SA-*β*-gal-positive cells. *n* = 3 per group. (c) IF staining for Ki-67 (red). DAPI was used to stain the nuclei. Scale bar, 50 *μ*m. (d) Percentage of Ki-67-positive cells. *n* = 3 per group. (e) The expression of P16 was assessed by western blotting. (f) Densitometric quantification of the relative band intensity in (e). *n* = 3 per group. (g) The expression of Collagen II, Aggrecan, MMP-3, and ADAMTS-4 was assessed by western blotting. (h) Densitometric quantification of the relative band intensity in (g). *n* = 3 per group. (i) Quantification of mRNA expression for SASP (TNF-*α*, IL-6, and IL-8). *n* = 3 per group. (^∗^*P* < 0.05 compared with IL-1*β* (-) and Adipo-sEV (-) group; ^#^*P* < 0.05 compared with IL-1*β* (+) and Adipo-sEV (-) group).

To explore the effect of Adipo-sEVs on senescent NPCs, we first established an in vitro model of cellular senescence by treating NPCs with 10 ng/mL IL-1*β* followed by the incubation of senescent NPCs with 1 × 10^10^ particles/mL Adipo-sEVs for seven days. As shown in Figure 2(a) and 2(b), NPCs treated with IL-1*β* exhibited increased numbers of SA-*β*-gal-positive cells; however, Adipo-sEV treatment significantly reduced this number. IF results showed that the proportion of Ki-67-positive cells in senescent NPCs increased after Adipo-sEV treatment (Figures 2(c) and 2(d)). The age-related P16 protein expression level in senescent NPCs was obviously downregulated by Adipo-sEV treatment (Figures 2(e) and 2(f)). Next, we evaluated the effects of Adipo-sEVs on several protein levels of the ECM in senescent NPCs. Western blot analysis demonstrated that Adipo-sEV treatment significantly restored the levels of collagen II and Aggrecan anabolism markers of ECM and suppressed the levels of MMP-3 and ADAMTS-4 catabolic markers of ECM in senescent NPCs (Figure 2(g) and 2(h)). The secretion of SASP in senescent NPCs, such as TNF-*α*, IL-6, and IL-8, was also reduced by Adipo-sEV treatment (Figure 2(i)). These results suggest that the senescent phenotype of NPCs and age-related dysfunction could be ameliorated by Adipo-sEV treatment.

### 3.3. Adipo-sEVs Can Ameliorate the Senescent Phenotypes of EPCs and Calcification

**Figure 3:**
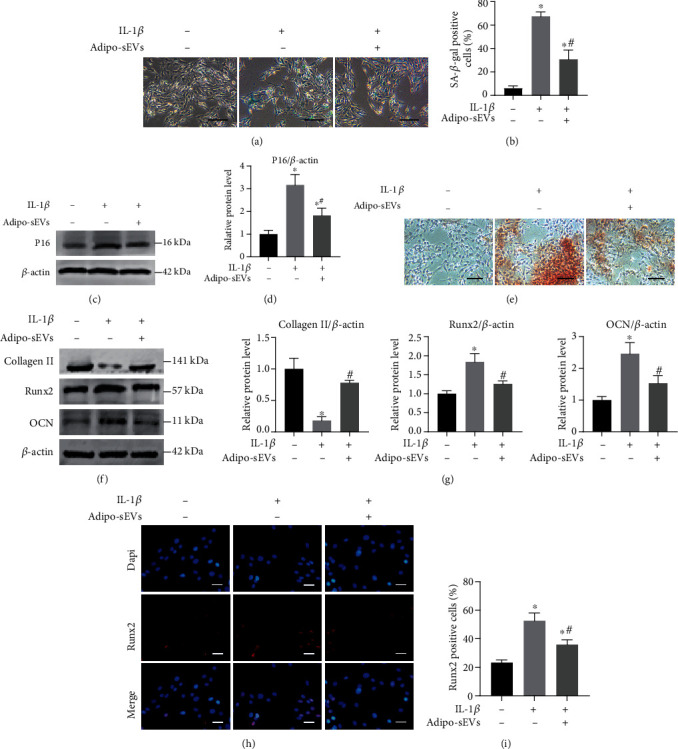
Adipo-sEVs can ameliorate the senescent phenotypes of EPCs and calcification. (a) Representative micrographs of EPCs stained with SA-*β*-gal in different treatment groups. SA-*β*-gal-positive cells are shown in blue. Scale bar, 100 *μ*m. (b) Percentage of SA-*β*-gal-positive cells. *n* = 3 per group. (c) The expression of P16 was assessed by western blotting. (d) Densitometric quantification of the relative band intensity in (c). *n* = 3 per group. (e) Representative micrographs of EPCs stained with Alizarin Red in different treatment groups. Scale bar, 100 *μ*m. Alizarin Red to detect mineralization. (f) Ossification-associated protein expression was assessed by western blotting. (g) Densitometric quantification of the relative band intensity in (f). *n* = 3 per group. (h) IF staining for Runx2 (red). DAPI was used to stain the nuclei. Scale bar, 50 *μ*m. (i) Percentage of Runx2-positive cells. *n* = 3 per group. (^∗^*P* < 0.05 compared with IL-1*β* (-) and Adipo-sEV (-) group; ^#^*P* < 0.05 compared with IL-1*β* (+) and Adipo-sEV (-) group).

Senescence of EPCs and accompanying cartilage calcification are also involved in the development of IVDD [5, 16]. Therefore, we examined the effect of Adipo-sEVs on senescent EPCs. We established a model of EPC senescence similar to that of NPCs. The results showed that the proportion of senescent EPCs (Figures 3(a) and 3(b)), as well as the P16 protein expression level (Figures 3(c) and 3(d)), increased after treating normal EPCs with 10 ng/mL IL-1*β*. Furthermore, we tested the calcification levels of the senescent EPCs. Alizarin Red staining showed that calcium synthesis was significantly increased in EPCs treated with IL-1*β* (Figure 3(e)). Ossification-associated protein expression, such as Runx2 and OCN, was consistently upregulated after IL-1*β* treatment (Figures 3(f) and 3(g)). We also detected Runx2 expression using IF. As shown in Figures 3(h) and 3(i), Runx2-positive cells significantly increased, analogous to the results of western blot analysis. However, the senescent phenotype of EPCs and increased calcification were dramatically reduced after the addition of 1 × 10^10^ particles/mL Adipo-sEVs for seven days (Figures 3(a) – 3(i)).

### 3.4. Adipo-sEVs Deliver NAMPT to Senescent NPCs and Enhance NAD^+^ and Sirt1 Activity

**Figure 4:**
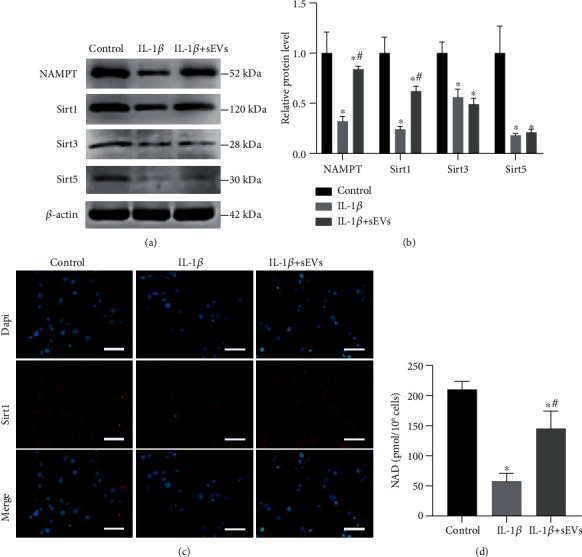
Adipo-sEVs deliver NAMPT into senescent NPCs and enhance NAD^+^ and Sirt1 activity. (a) Adipo-sEVs increased NAMPT and Sirt1 levels in senescent NPCs, but not Sirt3 and Sirt5 levels, as detected by western blotting. (b) Densitometric quantification of the relative band intensity in (a). *n* = 3 per group. (c) IF staining for Sirt1 (red). DAPI was used to stain the nuclei. Scale bar, 50 *μ*m. (d) NAD^+^ levels in NPCs treated with IL-1*β* and those cotreated with IL-1*β* and Adipo-sEVs. (^∗^*P* < 0.05 compared with the control group; ^#^*P* < 0.05 compared with the IL-1*β* group).

Adipo-sEV-mediated delivery of NAMPT can significantly mitigate tissue senescence and extend the lifespan of aged mice [12]. To investigate whether Adipo-sEVs can also transport NAMPT into senescent NPCs, we measured the level of NAMPT in senescent NPCs after the addition of Adipo-sEVs. Western blot analysis showed that senescent NPCs incubated with Adipo-sEVs exhibited higher levels of NAMPT compared to cells incubated with PBS (Figures 4(a) and 4(b)). However, an increase in intracellular NAMPT (iNAMPT) levels contributes to NAD^+^ biosynthesis and Sirt1/3/5 activity, which is critical for regulating the aging process [18–20]. Therefore, to determine which sirtuins are involved in the rejuvenation of senescent NPCs, Sirt1, Sirt3, and Sirt5 proteins were detected by western blotting. As shown in Figures 4(a) and 4(b), Adipo-sEV treatment significantly increased Sirt1 levels, while no changes were observed in Sirt3 and Sirt5 levels. Consistently, Sirt1 expression in IF was also significantly upregulated, analogous to that observed by western blotting (Figure 4(c)). To further confirm the effects of Adipo-sEV-containing NAMPT on intracellular NAD^+^ biosynthesis, we measured NAD^+^ levels and the results showed that Adipo-sEV treatment enhanced NAD^+^ levels in senescent NPCs (Figure 4(d)). These results provide compelling evidence that Adipo-sEVs deliver NAMPT to senescent NPCs and enhance NAD^+^ and Sirt1 activity.

### 3.5. Adipo-sEVs Deliver NAMPT to Senescent EPCs and Enhance NAD^+^ and Sirt1 Activity

**Figure 5:**
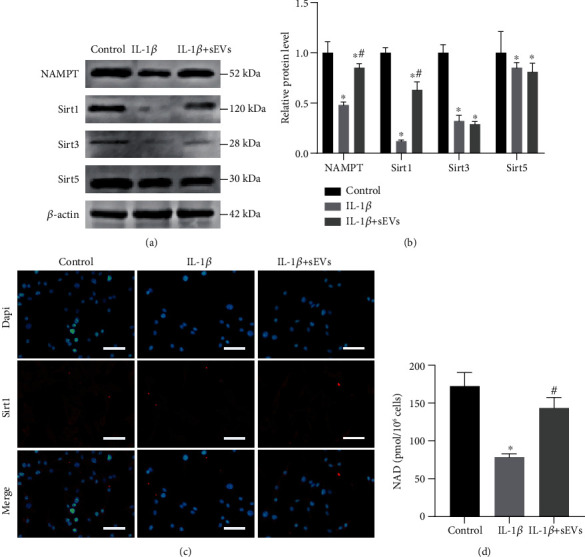
Adipo-sEVs deliver NAMPT into senescent EPCs and enhance NAD^+^ and Sirt1 activity. (a) Adipo-sEVs increased NAMPT and Sirt1 levels in senescent EPCs, but not Sirt3 and Sirt5 levels, as detected by western blotting. (b) Densitometric quantification of the relative band intensity in (a). *n* = 3 per group. (c) IF staining for Sirt1 (red). DAPI was used to stain the nuclei. Scale bar, 50 *μ*m. (d) NAD^+^ levels in EPCs treated with IL-1*β* and those cotreated with IL-1*β* and Adipo-sEVs. (^∗^*P* < 0.05 compared with the control group; ^#^*P* < 0.05 compared with the IL-1*β* group).

Having demonstrated that Adipo-sEVs rejuvenate senescent NPCs in an NAD^+^/Sirt1-dependent manner, we next examined whether Adipo-sEVs rejuvenated senescent EPCs in the same way. Western blot analysis showed that the addition of Adipo-sEVs enhanced the level of NAMPT in senescent EPCs as well as the level of Sirt1 (Figures 5(a) and 5(b)). IF of Sirt1 also showed an analogous result to that of the western blot (Figure 5(c)). NAD^+^ biosynthesis was elevated in senescent EPCs by Adipo-sEV treatment (Figure 5(d)). These results revealed that Adipo-sEVs rejuvenated senescent EPCs by NAD^+^/Sirt1 signalling.

### 3.6. sEVs Secreted from NAMPT-Knockdown Adipocytes Exhibit Attenuated Function on Rejuvenating Senescent NPCs

**Figure 6:**
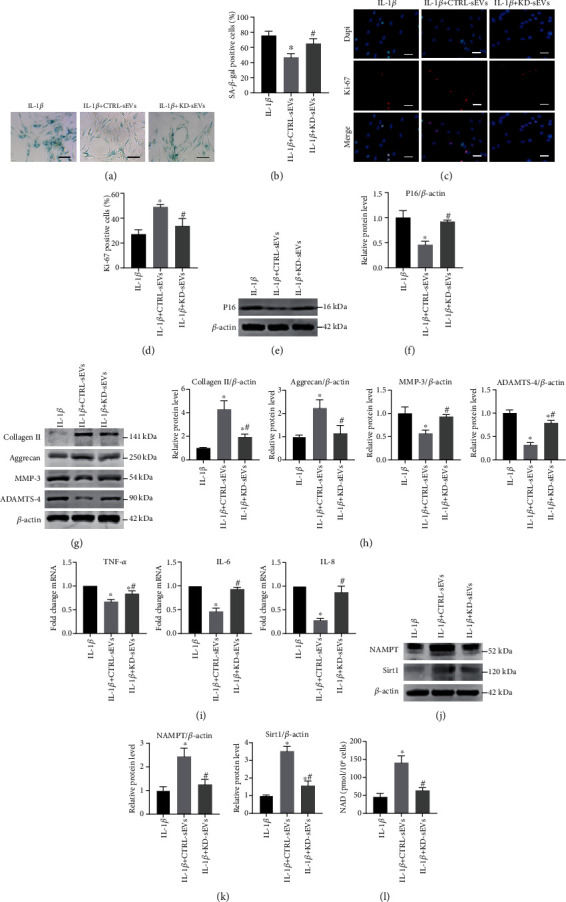
sEVs secreted from NAMPT-knockdown adipocytes exhibited attenuated function on rejuvenating senescent NPCs. (a) Representative micrographs of EPCs stained with SA-*β*-gal in different treatment groups. SA-*β*-gal-positive cells are shown in blue. Scale bar, 100 *μ*m. (b) Percentage of SA-*β*-gal-positive cells. *n* = 3 per group. (c) IF staining for Ki-67 (red). DAPI was used to stain the nuclei. Scale bar, 50 *μ*m. (d) Percentage of Ki-67-positive cells. *n* = 3 per group. (e) The expression of P16 was assessed by western blotting. (f) Densitometric quantification of the relative band intensity in (e). *n* = 3 per group. (g) The expression of Collagen II, Aggrecan, MMP-3, and ADAMTS-4 was assessed by western blotting. (h) Densitometric quantification of the relative band intensity in (g). *n* = 3 per group. (i) Quantification of mRNA expression for SASP (TNF-*α*, IL-6, and IL-8). *n* = 3 per group. (j) NAMPT and Sirt1 expression were assessed by western blotting. (k) Densitometric quantification of the relative band intensity in (j). *n* = 3 per group. (l) NAD^+^ levels in different treatment groups. (^∗^*P* < 0.05 compared with the IL-1*β* group; ^#^*P* < 0.05 compared with the IL-1*β* + CTRL-sEV group).

To confirm whether Adipo-sEV-containing NAMPT is crucial in Adipo-sEV-mediated rejuvenation of senescent NPCs, we compared the therapeutic effects of sEVs harvested from the conditioned medium of CTRL- and KD-sEVs. We first evaluated the NAMPT levels in KD- and CTRL-sEVs and found that NAMPT levels in KD-sEVs were significantly reduced compared to those in CTRL-sEVs (Supplementary Fig. 1). The following results demonstrated that KD-sEV treatment showed no reduction in the proportion of senescent NPCs, no restoration of proliferation capacity, and no downregulation of P16 protein levels (Figures 6(a) – 6(f)). Accordingly, CTRL-sEVs again exhibited significant increases in the levels of collagen II and Aggrecan and decreases in the levels of MMP-3, ADAMTS-4, and SASP. However, KD-sEVs failed to show these effects (Figures 6(g) – 6(i)). We also compared the effects of CTRL- and KD-sEVs on the levels of NAMPT and Sirt1 proteins and NAD^+^ biosynthesis in senescent NPCs. Western blot analysis showed that CTRL-sEVs enhanced the levels of NAMPT and Sirt1 proteins and promoted NAD^+^ biosynthesis in senescent NPCs, but KD-sEVs did not enhance the levels of NAMPT and Sirt1 proteins and NAD^+^ biosynthesis in senescent NPCs (Figures 6(j) – 6(l)), indicating that sEV-mediated delivery of NAMPT is key to NAD^+^/Sirt1 activity in senescent NPCs.

### 3.7. sEVs Secreted from NAMPT-Knockdown Adipocytes Exhibit Attenuated Function on Rejuvenating Senescent EPCs

**Figure 7:**
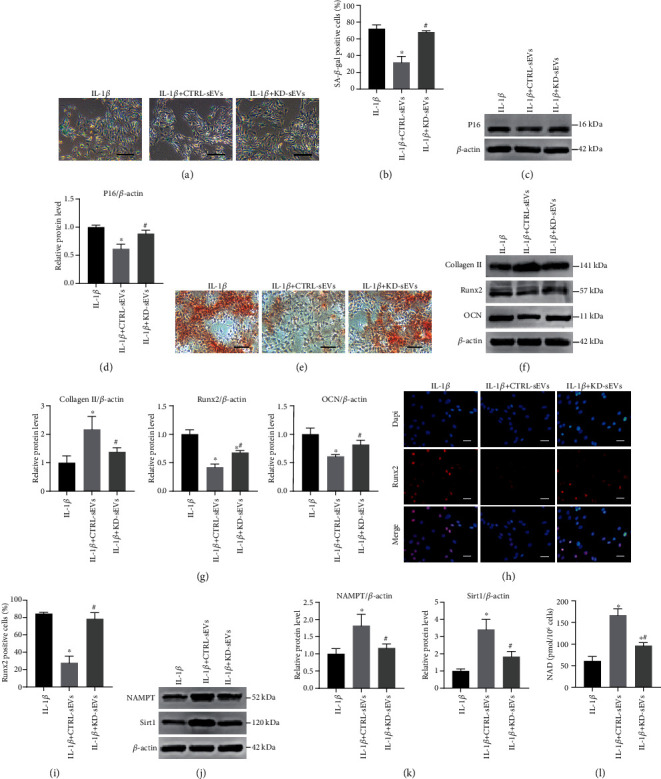
sEVs secreted from NAMPT-knockdown adipocytes exhibited attenuated function on rejuvenating senescent EPCs. (a) Representative micrographs of EPCs stained with SA-*β*-gal in different treatment groups. SA-*β*-gal-positive cells are shown in blue. Scale bar, 100 *μ*m. (b) Percentage of SA-*β*-gal-positive cells. *n* = 3 per group. (c) The expression of P16 was assessed by western blotting. (d) Densitometric quantification of the relative band intensity in (c). *n* = 3 per group. (e) Representative micrographs of EPCs stained with Alizarin Red in different treatment groups. Scale bar, 100 *μ*m. Alizarin Red to detect mineralization. (f) Ossification-associated protein expression was assessed by western blotting. (g) Densitometric quantification of the relative band intensity in (f). *n* = 3 per group. (h) IF staining for Runx2 (red). DAPI was used to stain the nuclei. Scale bar, 50 *μ*m. (i) Percentage of Runx2-positive cells. *n* = 3 per group. (j) The expression of NAMPT and Sirt1 was assessed by western blotting. (k) Densitometric quantification of the relative band intensity in (j). *n* = 3 per group. (l) NAD^+^ levels in different treatment groups. (^∗^*P* < 0.05 compared with the IL-1*β* group; ^#^*P* < 0.05 compared with the IL-1*β*+CTRL-sEV group).

Given that Adipo-sEV-contained NAMPT is crucial in the Adipo-sEV-mediated rejuvenation of senescent NPCs, we next explored whether this mechanism could also explain the effects of Adipo-sEVs on rejuvenating senescent EPCs. The results suggested that KD-sEVs failed to ameliorate senescent phenotypes of EPCs, including SA-*β*-Gal activity and P16 protein level (Figures 7(a) – 7(d)). In addition, the effects of Adipo-sEVs on calcification and ossification-associated protein expression in senescent EPCs were eliminated after the knockdown of NAMPT levels in Adipo-sEVs (Figures 7(e) – 7(i)). Similarly, we also evaluated the effects of CTRL- and KD-sEVs on the levels of NAMPT and Sirt1 proteins and NAD^+^ biosynthesis in senescent EPCs. Analogous to that in senescent NPCs, CTRL-sEVs enhanced the levels of NAMPT and Sirt1 protein and promoted NAD^+^ biosynthesis in senescent EPCs; however, these effects were not significantly observed after treating senescent EPCs with KD-sEVs (Figures 7(j) – 7(l)).

### 3.8. Inhibition of Sirt1 Attenuates the Therapeutic Effects of Adipo-sEVs on Senescent NPCs

**Figure 8:**
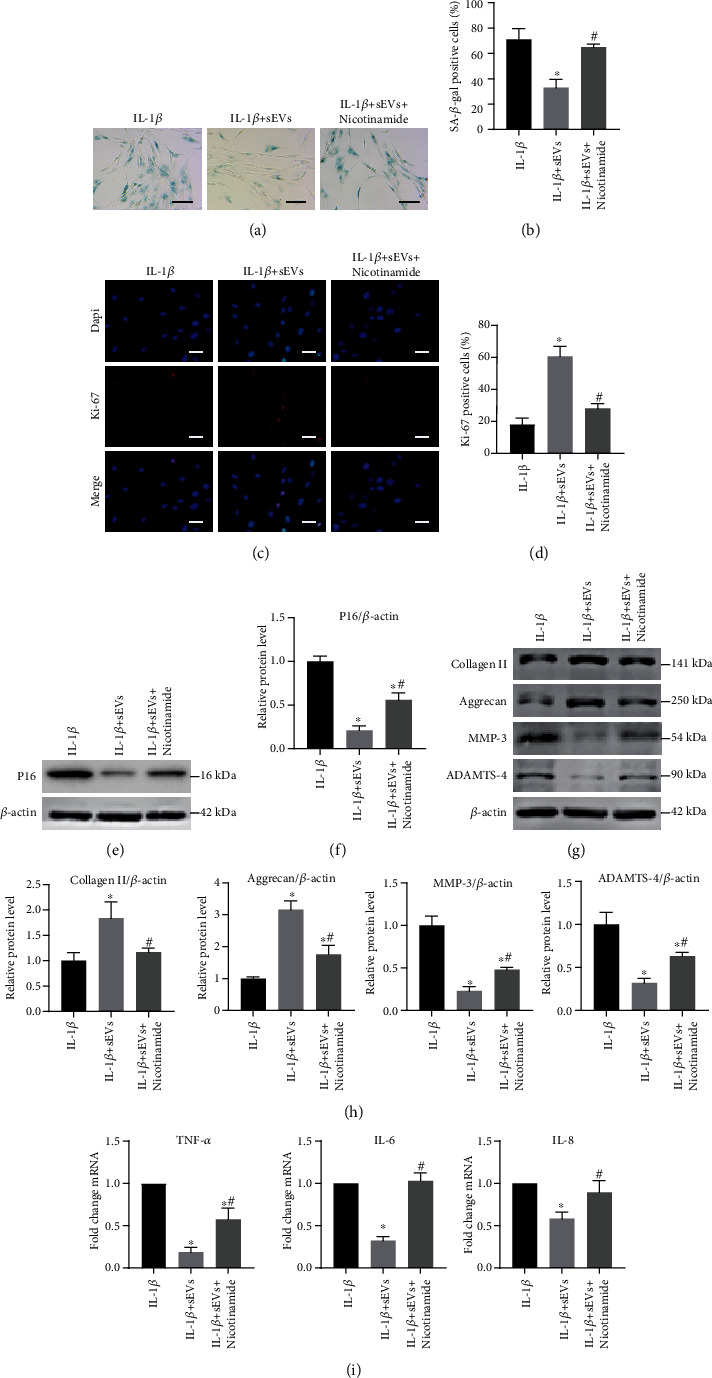
Inhibition of Sirt1 attenuates the therapeutic effects of Adipo-sEVs on senescent NPCs. (a) Representative micrographs of NPCs stained with SA-*β*-gal in different treatment groups. SA-*β*-gal-positive cells are shown in blue. Scale bar, 100 *μ*m. (b) Percentage of SA-*β*-gal-positive cells. *n* = 3 per group. (c) IF staining for Ki-67 (red). DAPI was used to stain the nuclei. Scale bar, 50 *μ*m. (d) Percentage of Ki-67-positive cells. *n* = 3 per group. (e) The expression of P16 was assessed by western blotting. (f) Densitometric quantification of the relative band intensity in (e). *n* = 3 per group. (g) The expression of Collagen II, Aggrecan, MMP-3, and ADAMTS-4 was assessed by western blotting. (h) Densitometric quantification of the relative band intensity in (g). *n* = 3 per group. (i) Quantification of mRNA expression for SASP (TNF-*α*, IL-6, and IL-8). *n* = 3 per group. (^∗^*P* < 0.05 compared with IL-1*β* group; ^#^*P* < 0.05 compared with the IL-1*β*+sEV group).

These results showed that Adipo-sEV treatment significantly increased the expression of Sirt1 in senescent NPCs rather than that of Sirt3 or Sirt5. Next, we aimed to verify whether Sirt1 signalling is crucial for the antiaging effect of Adipo-sEVs. We treated the senescent NPCs with Adipo-sEVs and 10 nM of nicotinamide (a Sirt1 inhibitor; Beyotime) for seven days and found that Adipo-sEVs failed to reduce the number of senescent cells, restore the Ki-67 expression, or downregulate the level of P16 (Figures 8(a) – 8(f)). In addition, the effects of Adipo-sEVs on metabolic markers of ECM in senescent NPCs, including an increase in anabolic markers and a decrease in catabolic markers, were blocked after the addition of nicotinamide (Figures 8(g) and 8(h)). The downregulation of SASP levels mediated by Adipo-sEVs was abolished by nicotinamide treatment (Figure 8(i)). These results demonstrate that Sirt1 signalling plays a key role in Adipo-sEV-mediated therapeutic effects of rejuvenating senescent NPCs.

### 3.9. Inhibition of Sirt1 Attenuates the Therapeutic Effects of Adipo-sEVs on Senescent EPCs

**Figure 9:**
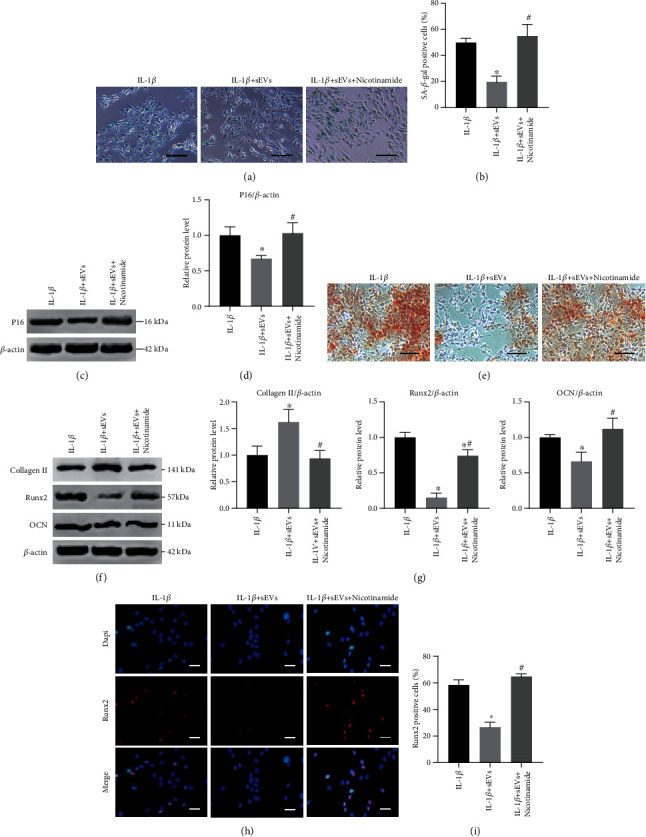
Inhibition of Sirt1 attenuates the therapeutic effects of Adipo-sEVs on senescent EPCs. (a) Representative micrographs of EPCs stained with SA-*β*-gal in different treatment groups. SA-*β*-gal-positive cells are shown in blue. Scale bar, 100 *μ*m. (b) Percentage of SA-*β*-gal-positive cells. *n* = 3 per group. (c) The expression of P16 was assessed by western blotting. (d) Densitometric quantification of the relative band intensity in (c). *n* = 3 per group. (e) Representative micrographs of EPCs stained with Alizarin Red in different treatment groups. Scale bar, 100 *μ*m. Alizarin Red to detect mineralization. (f) Ossification-associated protein expression was assessed by Western blotting. (g) Densitometric quantification of the relative band intensity in (f). *n* = 3 per group. (h) IF staining for Runx2 (red). DAPI was used to stain the nuclei. Scale bar, 50 *μ*m. (i) Percentage of Runx2-positive cells. *n* = 3 per group. (^∗^*P* < 0.05 compared with the IL-1*β* group; ^#^*P* < 0.05 compared with the IL-1*β*+sEV group).

Next, we examined whether Sirt1 upregulation contributes to the rejuvenating effects of Adipo-sEVs in senescent EPCs. Senescent EPCs were cotreated with Adipo-sEVs and nicotinamide for seven days. As shown in Figures 9(a) – 9(d), nicotinamide significantly attenuated Adipo-sEV-mediated decrease in the number of senescent cells, as well as the level of P16. The effects of Adipo-sEVs on calcification and ossification-associated protein expression in senescent EPCs were also dramatically abolished by nicotinamide treatment (Figures 9(e) – 9(i)). These results showed that Sirt1 signalling plays a key role in Adipo-sEV-mediated therapeutic effects of rejuvenating senescent EPCs and alleviating calcification.

### 3.10. Intradiscal Injection of Adipo-sEVs Attenuates the Development of IVDD in Rats by Rejuvenating Senescent NPCs and EPCs

**Figure 10:**
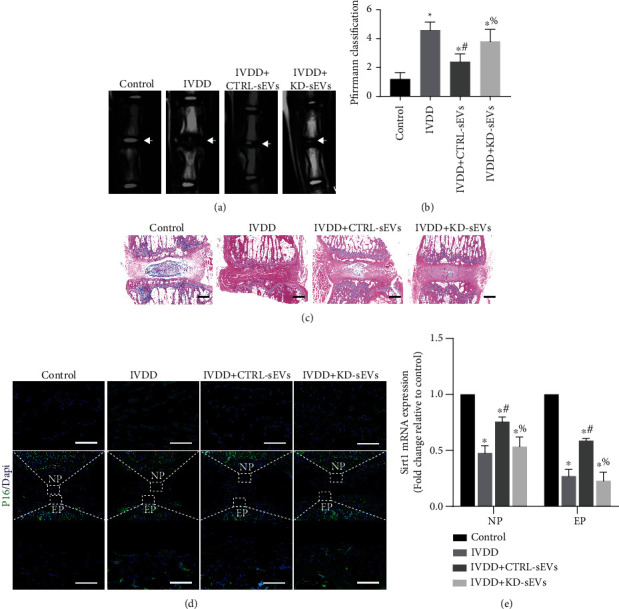
Intradiscal injection of Adipo-sEVs attenuated the development of IVDD in rats by rejuvenating senescent NPCs and EPCs. (a) MRIs of the indicated groups were obtained eight weeks after needle puncture. (b) Pfirrmann MRI grade scores. (c) H&E staining of IVDs in the indicated groups eight weeks after needle puncture. Scale bar, 1 mm. (d) IF staining for P16 (green) four weeks after needle puncture. DAPI was used to stain the nuclei. Scale bar, 1 mm. (e) Quantification of mRNA expression for Sirt1 in NP and EP. *n* = 3 per group. (^∗^*P* < 0.05 compared with the control group; ^#^*P* < 0.05 compared with the IVDD group; ^%^*P* < 0.05 compared with the IVDD+CTRL-sEV group).

MRIs indicated that the T2-weighted signal intensity in the IVDD group was weaker than that in the control group, and this change was improved by CTRL-sEV treatment. However, the therapeutic effect of Adipo-sEVs was eliminated by knockdown of NAMPT in Adipo-sEVs (Figure 10(a)). The IVDD score of MRI demonstrated significantly higher scores in the IVDD group than in the control group, and scores decreased markedly after Adipo-sEV treatment, while no significant improvement was observed using KD-sEVs (Figure 10(b)). Next, we evaluated the histological changes in the IVDs in each group by H&E staining. The results showed that CTRL-sEVs remarkably alleviated IVD degeneration and restored intervertebral height in a rat model of IVDD, while these improvements were significantly blocked after administering KD-sEVs (Figure 10(c)). IF staining for P16 revealed that CTRL-sEV intervention downregulated the P16 level in both NP and EP, and no significant improvement was observed using KD-sEVs (Figure 10(d)). In addition, we investigated the effect of Adipo-sEVs on the levels of Sirt1 in both NP and EP. The results showed that Sirt1 expression significantly decreased in both NP and EP in the IVDD group compared to that in the control group; however, CTRL-sEVs but not KD-sEVs restored Sirt1 expression in both NP and EP (Figure 10(e)). These results suggest that Adipo-sEV-mediated delivery of NAMPT could ameliorate senescence of NP and EP and attenuate IVDD by activating the Sirt1 signalling pathway in rats.

## 4. Discussion

Chronic inflammation has long been considered an important factor in IVDD progression. When the IVD is under sustained overloading, inflammatory factors in the microenvironment are chronically maintained at low concentrations. This pathological change usually leads to the damage of NPCs and EPCs, including DNA damage, NAD^+^ depletion, and the suspension of the cell cycle, which is recognised as cellular senescence [21]. Senescent NPCs showed impaired proliferation and the balance of ECM synthesis, especially a persistent proinflammatory phenotype called SASP, which influences the cellular microenvironment in a paracrine or autocrine manner [22, 23]. Given that the components of the NP are mainly determined by the secretion of NPCs, the decline in the function of senescent NPCs significantly affects the load-bearing and buffering of the spine. Improving NPC senescence can significantly promote matrix homeostasis and delay IVDD [24, 25]. However, EP is crucial for the IVD nutrient supply. Due to the lack of vascular structure in IVD, its nutrient supply is primarily dependent on the diffusion of nutrients through EP. Low but chronic inflammatory factors also contribute to the accumulation of senescent EPCs and perturb homeostasis of the EP matrix. Senescent EPCs exhibit increased calcification. Some studies have shown that pores in the bony EP are blocked [26], whereas others indicate that porosity in the bony EP increases [27, 28]. Either change results in an imbalance of nutrients and metabolites entering and exiting IVD and accelerates IVDD. Based on the above, the senescence of NPCs and EPCs has emerged as a central topic in the domain of IVDD research. How to effectively alleviate cellular senescence remains a challenge.

The results of the present study indicate that Adipo-sEV-mediated delivery of NAMPT attenuated IVDD in rats by rejuvenating senescent NPCs and EPCs. NAMPT is the rate-limiting enzyme that controls NAD^+^ biosynthesis, which is crucial for cellular energy metabolism and the regulation of various biological processes, such as aging [29]. NAMPT is responsible for the conversion of 5′-phosphoribosyl-pyrophosphate (PRPP) and nicotinamide to nicotinamide mononucleotide (NMN). There are two distinct forms of NAMPT in mammals: iNAMPT and extracellular NAMPT (eNAMPT) [30]. iNAMPT plays a key role in maintaining NAD^+^ synthesis; however, the function of eNAMPT remains controversial. Yoshida et al. found that the level of eNAMPT secreted by adipose tissue is correlated with physiological function in mice. They observed that plasma eNAMPT levels decline with age in mice and that supplementing eNAMPT in systemic circulation by overexpression of NAMPT in adipose tissue restores multiple tissue functions and extends the lifespan in aged mice [12]. NAMPT was originally thought to be produced in adipocytes, but now, it is believed to involve other tissues. eNAMPT is encapsulated in Adipo-sEVs and transported into target cells along with internalization of Adipo-sEVs, whereas the NAMPT protein cannot be internalized alone. The lipid bilayer structure of sEVs protects NAMPT and enables NAMPT to reach other tissues, especially in the tissues with low levels of NAMPT, to maintain their NAD^+^ biosynthesis. Although the specific targeting ability of NAMPT-containing sEVs to different tissues is still unclear, the sEV-mediated transfer of NAMPT between tissues represents a novel mechanism of intertissue communication. These eNAMPTs carried into senescent cells enhance NAD^+^ biosynthesis intracellularly and counteract age-associated pathophysiology. In this study, we also observed that sEVs secreted by HS-5 adipocytes were rich in NAMPT, and Adipo-sEV treatment significantly increased the levels of NAMPT in senescent NPCs and EPCs. Subsequent experiments demonstrated that NAD^+^ biosynthesis and Sirt1 activity in senescent NPCs and EPCs were promoted, while age-associated dysfunction was mitigated. The therapeutic potential of Adipo-sEVs on senescent NPCs and EPCs was eliminated by knockdown of NAMPT in Adipo-sEVs. It is worth noting that eNAMPT was first discovered as a proinflammatory cytokine, known as pre-B-cell colony-enhancing factor, but its proinflammatory effects have not yet been well-established. In the present study, no proinflammatory effects were observed.

sEVs may play an important role in intercellular communication by delivering proteins and miRNAs [31]. A previous research demonstrated that Adipo-sEVs could influence the phenotypes of distant tissues by transporting microRNAs [32]. However, the recent research has shown that Adipo-sEVs may also regulate the aging process in other tissues by transporting NAMPT protein [12]. In this context, we further explored the effect of Adipo-sEVs in other age-associated disease models. Our present results reveal the importance of Adipo-sEV-containing eNAMPT in rejuvenating senescent NPCs and EPCs, but the therapeutic effects of Adipo-sEVs on senescent cells were not entirely eliminated by knockdown of NAMPT, indicating that other biological components in Adipo-sEVs, such as microRNAs, may be involved in this process. Therefore, further investigations are necessary.

## 5. Conclusion

This is the first study, to our knowledge, to evaluate the therapeutic effects of Adipo-sEVs on IVDD. Adipo-sEVs rejuvenate senescent NPCs and EPCs by delivering NAMPT and activating NAD^+^ biosynthesis and the Sirt1 pathway, which is crucial for cellular energy metabolism and the aging process. Our results suggest the potential of a new cell-free tool and provide promising sEV-mediated delivery of NAMPT as a therapeutic approach for IVDD clinically.

## Data Availability

The datasets used and/or analyzed during the current study are available from the corresponding author on reasonable request.

## Supplementary Materials

Supplementary Materials Supplementary Figure 1: NAMPT levels in KD-sEVs were significantly reduced compared to those in CTRL-sEVs. (A) Levels of NAMPT and CD63 in CTRL-sEVs and KD-sEVs. (B) Densitometric quantification of the relative band intensity in (A). *n* = 3 per group (^∗^*P* < 0.05 compared with CTRL-sEVs).
