# Supplementary material for: Small Extracellular Vesicles Derived from Adipocytes Attenuate Intervertebral Disc Degeneration in Rats by Rejuvenating Senescent Nucleus Pulposus Cells and Endplate Cells by Delivering Exogenous NAMPT
Source: Oxid Med Cell Longev. 2021 Aug 14;2021:9955448. doi: 10.1155/2021/9955448 (PMC8382538; doi:10.1155/2021/9955448)
Supplement: Supplementary Materials — Supplementary Figure 1: NAMPT levels in KD-sEVs were significantly reduced compared to those in CTRL-sEVs. (A) Levels of NAMPT and CD63 in CTRL-sEVs and KD-sEVs. (B) Densitometric quantification of the relative band intensity in (A). n = 3 per group (∗P < 0.05 compared with CTRL-sEVs). [file 9955448.f1.docx]

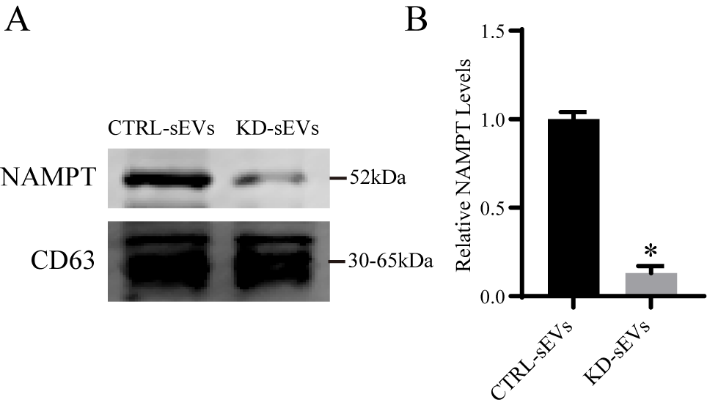


**Supplementary Figure 1. NAMPT levels in KD-sEVs were significantly reduced compared to those in CTRL-sEVs. (A)** Levels of NAMPT and CD63 in CTRL-sEVs and KD-sEVs. **(B)** Densitometric quantification of the relative band intensity in (A). n = 3 per group. (*P < 0.05 compared with CTRL-sEVs.)
